# Volumetric black-blood imaging of aortic dissection using T2 prepared inversion recovery

**DOI:** 10.1186/1532-429X-17-S1-P396

**Published:** 2015-02-03

**Authors:** Alia Noorani, Christoph Kiessewetter, Rene Botnar, Carlos A Figueroa, Markus Henningsson

**Affiliations:** 1 Division of Imaging Sciences and Biomedical Engineering, King's College London, London, UK; 2 Cardiothoracic Surgery, Papworth Hospital, Cambridge, UK; 3 Cardiology, Guy's and St. Thomas' NHS Foundation Trust, London, UK

## Background

Aortic dissection is a life threatening condition, and the commonest presentation of the acute aortic syndrome, occurring in 80-90% of such cases. Identification of intimal tears is important to enable the success of an interventional procedure. MRI allows for black-blood contrast to depict the aortic vessel wall and intimal flap with positive contrast which help delineate tear and intimal anatomy. However, the conventional black-blood approach (double inversion recovery) is limited to single slice imaging with relatively low resolution in slice direction. In this study, we compare the usefulness of two approaches using T2 prepared inversion recovery (T2IR) and T2 prepared phase sensitive inversion recovery (T2PSIR) for volumetric high-resolution black-blood aortic imaging in healthy volunteers including a pilot study to optimize flow-independence.

## Methods

A pilot study to investigate the flow-dependence of T2prep pulses and a volunteer study comprising of 11 healthy subjects was performed on a 3T MR scanner (Achieva, Philips Healthcare, NL) comparing T2PSIR and T2IR black blood sequences*.* The two sequences were then compared by means of a visual assessment score (VS, 1-4, 1 = poor image quality and 4= excellent image quality), contrast-to-noise ratio (CNR), image sharpness and scan time. For the pilot study one scan was acquired with the T2prep pulse applied in mid systole (high flow) and another scan with the T2prep pulse applied in early systole (low flow). Additionally, these sequences were tested on two patients with known chronic aortic dissection who were under a surveillance program.

## Results

The pilot study demonstrated the flow-dependence of the T2PSIR sequence. However, positioning the T2prep in a low flow cardiac phase (mid-diastole) minimizes flow artifacts. In the 11 healthy volunteers the median of difference in visual scores between the sequences was 2. Although the T2PSIR sequence was on average nearly twice as long as the T2IR sequence (median 1.9 ±0.45 times, mean time for T2PSIR = 703.7s ±210s vs.443.2s ±251.7s for T2IR) it was three times clearer than the T2IR sequences based on the CNR and (mean CNR for T2PSIR=25 ±7.5 vs.9.19 ±5.6 for T2IR) 1.7 times sharper as based on percentage sharpness (mean 74.1%±14.1% for T2PSIR vs.T2IR 46.9% ±13.4%). The VS for T2PSIR were better than T2IR in ten out of eleven cases, with one case scoring a similar result.

## Conclusions

In this study of vessel wall analysis comprising of eleven healthy volunteers, the T2PSIR sequence although was a longer scan, generated images of a superior quality as measured objectively and subjectively compared to the T2IR sequence. Subsequent application of the T2PSIR sequence in two patients with chronic aortic dissection demonstrated the intimal flap in excellent detail. Further application of this sequence in imaging of aortic diseases in particular dissection may help treatment planning as the quality of images obtained may provide detailed tear and flap morphology.

## Funding

This work was supported by research fellowships from the Royal College of Surgeons of England, UK and Heart Research UK.

**Figure 1:**
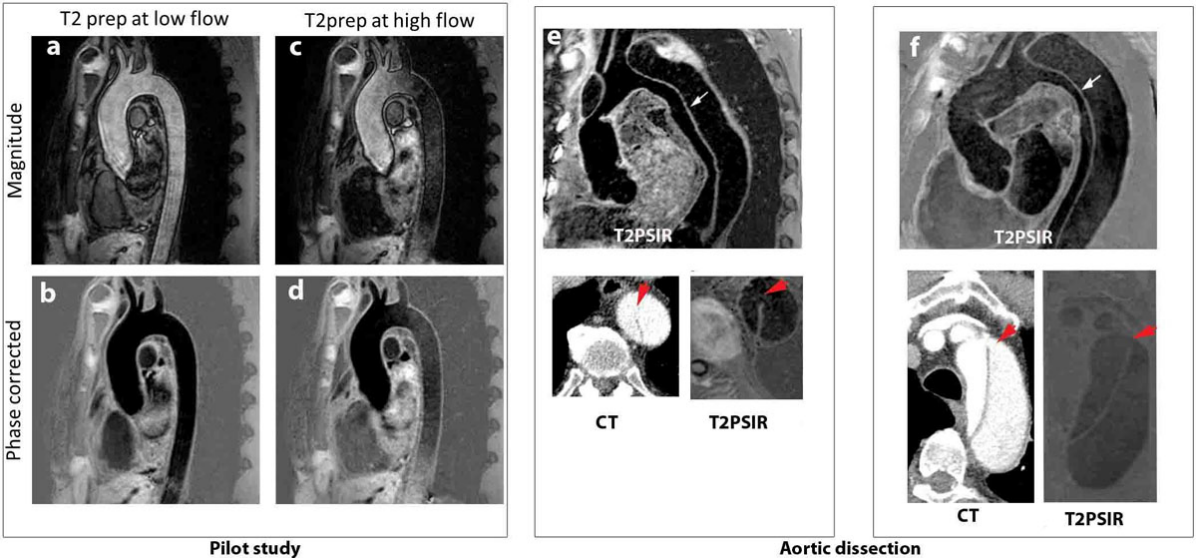
**Pilot study results demonstrate flow artifacts if the T2 prep is positioned in a high flow phase (c, d) which are avoided by performing the T2prep in a low flow phase (a, b).** Two patients with aortic dissection scanned with the T2PSIR sequence, which shows the intimal tear and flap clearly (e, f). Corresponding CT slices showing the tear and intimal flap are included for comparison.

